# The professional competence of newly graduated nurses in the transition phase as assessed by nurse managers: a descriptive cross-sectional multi-national study

**DOI:** 10.1177/17449871241311543

**Published:** 2025-03-15

**Authors:** Pia Kukkonen, Sanna Koskinen, Pilar Fuster-Linares, Natalja Istomina, Helena Leino-Kilpi, Eliisa Löyttyniemi, Gabriele Meyer, Leena Salminen, Herdís Sveinsdóttir, Asta Heikkilä

**Affiliations:** Doctoral Candidate, Department of Nursing Science, University of Turku, Turku, Finland; University Teacher, Department of Nursing Science, University of Turku, Turku, Finland; Lecturer, Department of Nursing, International University of Catalonia, Barcelona, Spain; Professor, Head of Department of Nursing, Vilnius University, Lithuania; Professor (emerita), Researcher, Department of Nursing Science, University of Turku, Turku, Finland; Turku University Hospital/Wellbeing Services County of Southwest Finland, Finland; Biostatistician, Department of Biostatistics, University of Turku and Turku University Hospital/Wellbeing Services County of Southwest Finland, Finland; Professor, Institute of Health and Nursing Science, Martin Luther University Halle-Wittenberg, Germany; Professor (emerita), Department of Nursing Science, University of Turku; Turku University Hospital/Wellbeing Services County of Southwest Finland, Finland; Professor, Faculty of Nursing, University of Iceland, Iceland; Clinical Lecturer, Director of Nursing Excellence, Department of Nursing Science, University of Turku Turku University Hospital/Wellbeing Services County of Southwest Finland, Finland

**Keywords:** newly graduated nurse, nurse manager, professional competence, transition phase

## Abstract

**Background:**

Nurse managers’ (NMs) assessment of nurses’ competences is needed to analyse how well the educational preparation corresponds with the requirements of nursing practice in Europe.

**Aim:**

To assess newly graduated nurses’ professional competence in the transition phase as perceived by NMs and to identify possible background factors related to their assessments.

**Methods:**

A descriptive cross-sectional multinational study. Data were collected in 2019 from NMs (*n* = 425) in Finland, Germany, Iceland, Lithuania and Spain using the structured Nurse Competence Scale and statistically analysed.

**Results:**

NMs assessed the level of newly graduated nurses’ competence as ‘good’. However, the overall competence varied between different countries. In all countries, the subcategory ‘Managing situations’ scored the highest and ‘Therapeutic interventions’ the lowest. NMs’ background factors were related to their assessment.

**Conclusions:**

Newly graduated nurses were assessed to have a good level of professional competence to meet the demands of their work in the transition phase, although there is room for improvement. The results can be used for cooperation between working life and nursing education to identify areas where the professional competence of newly qualified nurses can be improved and to promote their transition and continuous professional development in Europe.

## Introduction

In recruitment, an important role and responsibility of nurse managers (hereafter NM) are to ensure that newly graduated nurses (NGNs) are sufficiently competent and meet the requirements of working life to provide safe, high-quality, effective and evidence-based care to patients (Brown and Crookes, 2016; Julnes et al., 2022) and to work effectively within the organisation (Aiken et al., 2014). In this study, NMs are defined as leaders working in middle management or strategic leadership roles in healthcare organisations, with a focus on collaboration, human resource management, operational management, ensuring staff competence and, in different ways, clinical care (González-García et al., 2021; Warhaswsky and Cramer, 2019). The study focuses on the stage at which NGNs move from the role of student to a responsible professional. Transition phase typically refers to the first year in practice (Duchscher, 2008) and therefore, in this study, NGNs were defined as qualified nurses who had graduated a maximum of one year ago. At this stage, the link to nursing education is apparent, and it is also the starting point for the NGNs’ career development.

The study was undertaken across five European. The international data collection is based on the fact that the shortage of nurses is global, and many countries have increased enrolment in nursing education (Ma et al., 2023; OECD, 2023). There has been an attempt to harmonise nursing education in Europe and at least up to a certain point, there are convergent nursing programmes (European Union [EU], 2005/36/EC, 2013/55EU). Despite the common ground, there are differences in nursing education (Antao et al., 2023; Eronen et al., 2023; Henriksen et al., 2020), and challenges have been reported in relation to NGNs’ professional competence and transition to practice (Masso et al., 2022). This study focuses on assessing the professional competence of NGNs in transition phase as perceived by NMs. Studying professional competence of NGNs allows to analyse how well the educational preparation corresponds with the requirements of nursing practice in Europe and to identify possible areas for development.

## Background

In this study, professional competence of NGNs is defined as ‘functional adequacy and capacity to integrate knowledge and skills to attitudes and values into specific contextual situations for practice’ (Meretoja et al., 2004a: 330–331). According to this definition, the professional competence of nurses is expressed through the following activities: helping, teaching and coaching, diagnostic functions, managing situations, therapeutic interventions, ensuring quality and work role (Flinkman et al., 2017; Lejonqvist and Kajander-Unkuri, 2022; Meretoja et al., 2004b). The terms competence and competency are used synonymously, although the nursing literature distinguishes between them (Khan and Ramachandran, 2012; Pijl-Zieber et al., 2014).

In this study, NMs assess the professional competence of NGNs. The assessor has an important role in determining the level of competence of the NGN, as it is related to different factors and also varies depending on the assessor (Hyun et al., 2020; Kajander-Unkuri et al., 2020; Numminen et al., 2014). Thus, mentors are often more critical in their assessments compared to graduating nursing students themselves (Kajander-Unkuri et al., 2016), while nurse educators tend to rate the competence of NGNs significantly higher than NMs (Numminen et al., 2014). These assessments may vary because nurse educators and NMs have a different reference point for the level of competence required (Kukkonen et al., 2020; Numminen et al., 2014). There is earlier research on the competence of nurses as assessed by NMs (Bahreini et al., 2011; Meretoja & Leino-Kilpi, 2003; Numminen et al., 2015; Önal and Intepeler, 2024). However, the viewpoint of NMs on NGNs’ professional competence is seldom studied, primarily at the national level (Kukkonen et al., 2020), and the results have mainly been reported together with different participant groups such as educators (Hyun et al., 2020; Numminen et al., 2014) and nurse preceptors (Gregg, 2020). NMs’ competence assessment is needed as an indicator of the work performance of the NGNs and to bridge the gap between working life and nursing education (Gregg, 2020) by optimising conditions for NGNs’ transition to practice and by ensuring their ongoing competence and career development (Brown and Crookes, 2016; Hyun et al., 2022; Ma et al., 2023; Södersved Källestedt et al., 2020).

Previous studies with NMs have primarily concentrated on assessing the observable competency of NGNs, whereas recent research has evaluated professional competence more comprehensively, across various domains. NMs’ assessments vary without any consensus (Kukkonen et al., 2020, 2023). NMs have generally rated the competence of NGNs as quite good in competence categories such as ‘helping’, ‘ensuring quality’, ‘teaching/coaching’, ‘diagnostic functions’ and ‘managing situations’ (Numminen et al., 2014), and NGNs are reported to be able to demonstrate the majority of the competencies learned during nursing education (Charette et al., 2019a). When looking in more detail at specific competencies, NGNs have been reported to be well prepared to show respect and empathy for patients (Hyun et al., 2020; Serafin et al., 2020), to commit to ethical values (Numminen et al., 2014), to work as part of a team (Hopkins and Bromley, 2016), in utilising information technology (Numminen et al., 2014) and to be open to development and knowledge (Serafin et al., 2020). However, the assessments of NMs also show needs for competence development in areas such as decision-making (Serafin et al., 2020), emergency care (Hyun et al., 2020), main clinical areas (Kukkonen et al., 2020), coaching, guiding and mentoring tasks (Numminen et al., 2014), communication with interprofessional team (Hopkins and Bromley, 2016; Serafin et al., 2020), leadership skills (Södersved Källestedt et al., 2020), teamwork (Serafin et al., 2020) and ability to use knowledge in practice (Serafin et al., 2020). However, different descriptions of competence and assessment methods make it challenging to generalise and compare the findings (Kukkonen et al., 2020).

There is a lack of research on factors related NMs’ assessments of NGNs professional competence. NMs’ assessments of novice nurses’ competence have been found to be related to their age and work experience (Numminen et al., 2014). Moreover, Kukkonen et al. (2023) found that NMs’ assessments of graduating nursing students’ competence are related to the country, managers’ level of postgraduate education and their satisfaction with the nursing degree programme.

To conclude, nursing education in Europe is aiming to produce generic professional competencies (EU, 2005, 2013). The assessment of NGNs professional competence in transition phase support analysis of how well the educational preparation meets the requirements of working life and identifies possible areas for improvement. Research on this topic is lacking, and empirical studies are needed that comprehensively assess NGNs’ professional competence from the perspective of NMs’ (Kukkonen et al., 2020), including international comparisons. Such research is important as cross-border mobility of healthcare professionals affects the quality and safety of patient care across Europe.

## Aim

This multinational European study aims to assess NGNs’ professional competence in the transition phase as related to NMs’ assessment and to identify possible background factors related to their assessments. The assessment is carried out by NMs because they are expected to have a good understanding of the qualifications required in the workplace. The goal is to provide knowledge for collaboration between registered practice and nurse education to promote the transition and continuous professional competence development of the NGNs.

## Methods

### Study design

The design of this study was a descriptive cross-sectional multi-country study reported according to the STROBE statement (Von Elm et al., 2008). This study is part of the large international ProCompNurse research project interested in the competence and quality of the nursing workforce in Europe.

### Setting

Data were collected from NMs across 33 hospital organisations. The survey covered Finland, Germany, Iceland, Lithuania and Spain representing Northern, Southern and Central Europe. In Finland, Iceland and Lithuania, participating hospitals were located across the country, whereas in Germany and Spain, data were collected regionally. All the countries are members of the European Higher Education Area (EHEA), and the education is conducted according to the Bologna process and EU directives (EHEA, 2024; EU, 2005, 2013).

### Participants

All levels of nursing management were targeted to participate in this study, from unit level (NMs and assistant NMs) to middle and strategic level of management (nurse directors and executive nurses). Convenience sampling was used. Potential participants who met the inclusion criteria were identified by the national research teams in collaboration with the designated contact person at each participating hospital. The inclusion criteria for NMs were: (1) background as a nurse, (2) in a supervisory position, (3) connections to the nursing staff on a daily or almost daily basis and (4) contributes to, or is responsible for, the recruitment of nursing staff. Altogether 425 managers participated in this study giving the response rate of 67.4%.

### Data collection

In each participating country, there was a named national coordinator responsible for the data collection according to the study protocol. Data were collected in each country between 1st November and 15th December 2019. Two reminders were sent. Paper-and-pencil format was mainly used for data collection, except for Germany where electronic format was used. For paper-and-pencil format, the information letter and questionnaires were delivered to potential respondents in a sealed envelope either by hand or via post by a contact person. The questionnaires were returned either by post in a prepaid envelope to the national research team or collected from organisations from the appointed place in the unit. For the electronic format in Germany, by applying SoSci Survey software, local contact persons sent managers a link to their work email address to participate in the survey. NMs responded the questionnaire at a time suitable for them and lastly, sent in their responses via the software.

### Questionnaire

The structured questionnaire included two parts. The first part contained multiple-choice questions regarding NMs’ individual (age, gender, undergraduate education, postgraduate degree, work experience as a manager, work experience in the current unit) and organisational (current work title, staff report, NGNs seeking to work in unit and managers’ satisfaction with the current orientation programme, current job, the quality of care in the unit and nursing profession) background factors (Table 1). The second part of the questionnaire contained the Nurse Competence Scale (NCS, Meretoja et al., 2004a) which is an instrument allowing generic professional competence assessment between different countries, organisations and a variety of settings (Flinkman et al., 2017; Lejonqvist and Kajander-Unkuri, 2022). The NCS contains 73 items in seven theoretical categories: helping role (7 items), teaching-coaching (16 items), diagnostic functions (7 items), managing situations (8 items), therapeutic interventions (10 items), ensuring quality (6 items) and work role (19 items). Managers assessed the NGNs’ competence in the transition phase using a visual analogue scale: scores < 25 indicated ‘a low competence’, ⩾25–50 ‘quite good competence’, >50–75 ‘good competence’ and >75–100 ‘very good competence’ (Meretoja et al., 2004a; Flinkman et al., 2017). The option ‘not applicable’ was also available. The NCS was already validated and available in the target language for each participating country (Kukkonen et al., 2023). In this study, the Cronbach’s alpha values ranged from 0.92 to 0.97 across the categories indicating appropriate internal consistency (DeVellis, 2012). A double-translation process was conducted on the background factors with missing translations (Sousa and Rojjanasrirat, 2011).

**Table 1.** Background of the nurse managers (*n* = 425).

Background of managers	All, *N* = 425 Median (Q1;Q3) or *n* (%)	Finland, N = 112 Median (Q1;Q3) or *n* (%)	Germany, *N* = 59 Median (Q1;Q3) or *n* (%)	Iceland, *N* = 15 Median (Q1;Q3) or *n* (%)	Lithuania, *N* = 110 Median (Q1;Q3) or *n* (%)	Spain *N* = 129 Median (Q1;Q3) or *n* (%)
Individual background factors						
Age (years)	47.0 (41.0; 55.0)	53.0 (45.0; 59.0)	47.0 (34.0; 52.0)	51.0 (43.0; 53.0)	47.0 (42.0; 57.0)	43.0 (39.0; 49.0)
Gender						
Female	369 (87.0)	107 (96.4)	44 (74.6)	15 (100.0)	109 (99.1)	94 (72.9)
Male	55 (13.0)	4 (3.6)	15 (25.4)	0 (0.0)	1 (0.9)	35 (27.1)
Undergraduate nursing education						
University degree (bachelor)	222 (52.4)	42 (37.5)	2 (3.4)	12 (80.0)	48 (44.0)	118 (91.5)
College/Diploma	171 (40.3)	66 (58.9)	48 (81.4)	2 (13.3)	46 (42.2)	9 (7.0)
Other	31 (7.3)	4 (3.6)	9 (15.3)	1 (6.7)	15 (13.8)	2 (1.6)
Postgraduate degree						
Doctoral degree	14 (3.3)	3 (2.8)	0 (0.0)	0 (0.0)	4 (3.7)	7 (5.4)
Master’s degree	157 (37.5)	40 (37.4)	4 (6.8)	5 (33.3)	34 (31.2)	74 (57.4)
Bachelor’s degree	119 (28.4)	4 (3.7)	13 (22.0)	10 (66.7)	48 (44.0)	44 (34.1)
No postgraduate degree	129 (30.8)	60 (56.1)	42 (71.2)	0 (0.0)	23 (21.1)	4 (3.1)
Work experience as a manager (years)	9.2 (3.5; 17.0)	10.2 (4.8; 14.5)	8.0 (4.0; 21.0)	8.3 (2.8; 15.0)	16.0 (6.8; 25.3)	6.0 (2.0; 11.0)
Work experience on the current unit (years)	6.5 (2.0; 13.8)	5.3 (1.8; 12.0)	7.0 (2.5; 13.0)	9.0 (2.8; 19.2)	12.0 (4.5; 20.0)	3.0 (1.5; 10.0)
Organisational background factors						
Current work title						
Director of Nursing	16 (3.9)	9 (8.0)	0 (0.0)	0 (0.0)	4 (4.0)	3 (2.4)
Nurse Unit Manager	91 (22.1)	60 (53.6)	8 (13.6)	14 (100.0)	3 (3.0)	6 (4.8)
Assistant Unit Manager	253 (61.4)	42 (37.5)	50 (84.8)	0 (0.0)	76 (75.3)	85 (67.5)
Other	52 (12.6)	1 (0.9)	1 (1.7)	0 (0.0)	18 (17.8)	32 (25.4)
Staff report	35.0 (22.0; 60.0)	35.0 (28.0; 56.0)	28.0 (20.0; 46.0)	50.0 (40.0; 60.0)	24.5 (16.0; 35.0)	54.5 (30.0; 100.0)
NGNs seeking to work in managers’ unit						
Very seldom	45 (10.9)	7 (6.3)	4 (6.8)	2 (14.3)	23 (22.3)	9 (7.2)
Seldom	88 (22.3)	10 (8.9)	19 (32.2)	6 (42.9)	33 (32.0)	20 (16.0)
Often	213 (51.6)	66 (58.9)	28 (47.5)	5 (35.7)	40 (38.8)	74 (59.2)
Very often	67 (16.2)	29 (25.9)	8 (13.6)	1 (7.14)	7 (6.8)	22 (17.6)
Satisfaction with. . .						
the current orientation programme in unit						
Fully disagree	10 (2.4)	4 (3.6)	1 (1.7)	0 (0.0)	2 (1.9)	3 (2.4)
Almost disagree	48 (11.6)	13 (11.6)	14 (23.7)	3 (23.1)	6 (5.8)	12 (9.5)
Almost agree	237 (57.3)	69 (61.6)	26 (44.1)	8 (61.5)	55 (52.9)	79 (62.7)
Fully agree	119 (28.7)	26 (23.2)	18 (30.5)	2 (15.4)	41 (39.4)	32 (25.4)
Current job						
Fully disagree	3 (0.7)	0 (0.0)	1 (1.7)	0 (0.0)	2 (1.9)	0 (0.0)
Almost disagree	30 (7.2)	11 (10.0)	8 (13.6)	0 (0.0)	8 (7.5)	3 (2.4)
Almost agree	212 (51.1)	61 (55.5)	27 (45.8)	7 (53.9)	46 (43.0)	71 (56.4)
Fully agree	170 (41.0)	38 (34.6)	23 (39.0)	6 (46.2)	51 (47.7)	52 (41.3)
The quality of care in unit						
Fully disagree	6 (1.5)	2 (1.8)	1 (1.7)	0 (0.0)	2 (1.9)	1 (0.8)
Almost disagree	16 (3.9)	2 (1.8)	7 (11.9)	0 (0.0)	1 (0.9)	6 (4.8)
Almost agree	248 (60.0)	66 (60.0)	44 (74.6)	8 (61.5)	59 (55.1)	71 (56.8)
Fully agree	144 (34.8)	40 (36.4)	7 (11.9)	5 (38.5)	45 (42.1)	47 (37.6)
the nursing profession						
Fully disagree	4 (1.0)	1 (0.9)	1 (1.7)	0 (0.0)	2 (1.9)	0 (0.0)
Almost disagree	26 (6.3)	5 (4.6)	16 (27.1)	0 (0.0)	4 (3.7)	1 (0.8)
Almost agree	198 (47.7)	51 (46.4)	31 (52.5)	6 (46.2)	39 (36.5)	71 (56.4)
Fully agree	187 (45.1)	53 (48.2)	11 (18.6)	7 (53.9)	62 (57.9)	54 (42.9)

Q1: lower quartile; Q3: upper quartile.

### Data analysis

Continuous variables are summarised with mean (with standard deviation, SD) or median together with lower (Q1) and upper quartile (Q3) and categorical variables with counts and percentages.

Comparisons between countries for overall NCS and subscores were performed with one-way analysis of variance (ANOVA). Tukey’s correction method was used for multiple comparisons. Firstly, univariate modelling to NCS (overall and subscores) was performed with two-way analysis of variance or covariance (ANOVA/ANCOVA) for the most relevant background variables and including the study country in each model. Then, a multivariable model was built up with those explanatory variables which were significant in the univariate approach and did not have strong association with another background variable. Where country was associated with many background variables causing collinearity problems, it was left out of multivariable modelling. If the response or the value of any of the explanatory variables was missing, the participant was automatically removed from that analysis. However, the amount of missing data was very low. From these models, model-based means and slope estimates together with 95% confidence intervals are reported. The assumption of these models was checked using studentised residuals.

*p*-values less than 0.05 (two-tailed) were considered statistically significant. Data analysis was generated using SAS software, Version 9.4 of the SAS System for Windows (SAS Institute Inc., Cary, NC, USA).

## Results

### Background of the NMs

NMs (*n* = 425) from five different European countries participated in this study: Finland (*n* = 112), Germany (*n* = 59), Iceland (*n* = 15), Lithuania (*n* = 110) and Spain (*n* = 129). The median age of the NMs was 47 years and most were women. In the case of undergraduate nursing education, the NMs had graduated from different types of education. In three participating countries, the majority of the NMs had a postgraduate degree. The mean length of work experience as a NM was 9.2 years, the current work title for most (61.4%) was assistant unit manager, and they had on average 35.0 subordinates. The majority of the NMs (67.8%) responded that NGNs seek to work in their unit often or very often. Overall, the NMs almost or fully agreed that they were satisfied with their job (92.1%), the nursing profession (92.8%), quality of care in the unit/ward (94.8%) and the current orientation programme in their unit (86.0%; Table 1.)

### Professional competence of NGNs assessed by NMs

NMs assessed NGNs’ overall professional competence level with NCS (Meretoja et al., 2004a) as good (mean 58.2; SD 20.4; Table 2). The overall competence varied statistically significantly between different countries from 45.2 (SD 17.5) ‘quite good’ competence in Finland to 75.8 (13.7) ‘very good competence’ in Lithuania. Competence level in all seven competence categories was also assessed as good even though there were differences between countries. Among all countries, the category ‘Managing situations’ scored highest (mean 60.6) and ‘Therapeutic interventions’ lowest (55.0). In single countries, the category ‘Managing situations’ scored highest in Lithuania (82.2) and Germany (71.9), whereas ‘Diagnostic functions’ in Iceland (62.4), ‘Work role’ in Spain (55.1) and ‘Helping role’ in Finland (52.5) scored highest. The lowest scored categories were ‘Teaching-coaching’ in Lithuania (68.9) and Iceland (49.4), ‘Ensuring quality’ in Germany (56.4) and ‘Therapeutic interventions’ in Spain (47.9) and Finland (40.8; Table 2).

**Table 2.** Newly graduated nurses’ professional competence as assessed by nurse managers (*n* = 425).

Professional competence (Meretoja et al., 2004a)	All Mean (SD)	Finland Mean (SD)	Germany Mean (SD)	Iceland Mean (SD)	Lithuania Mean (SD)	Spain Mean (SD)	*p*-Value^1^
Overall competence (73 items)	58.2 (20.4)	**45.2**^2^ (17.5)	65.5^3^ (18.5)	56.7^4^ (17.6)	**75.8**^5^ (13.7)	52.2 (17.2)	<0.0001
Helping role (7 items)	59.6 (19.0)	52.5 (16.4)	64.7 (19.0)	60.5 (15.8)	73.5 (15.3)	53.4 (17.8)	<0.0001
Teaching-coaching (16 items)	55.8 (19.8)	47.6 (17.4)	63.1 (19.8)	49.4 (16.3)	68.9 (17.1)	51.7 (18.3)	<0.0001
Diagnostic functions (7 items)	59.3 (21.4)	47.6 (18.3)	69.4 (18.7)	62.4 (20.0)	74.3 (17.6)	52.9 (19.1)	<0.0001
Managing situations (8 items)	60.6 (24.1)	42.2 (19.8)	71.9 (20.0)	59.8 (19.6)	82.2 (14.7)	54.1 (19.7)	<0.0001
Therapeutic interventions (10 items)	55.0 (24.1)	40.8 (19.6)	59.5 (22.2)	53.5 (19.7)	76.9 (15.6)	47.9 (21.7)	<0.0001
Ensuring quality (6 items)	55.9 (22.6)	46.6 (21.7)	56.4 (22.9)	52.2 (20.6)	72.4 (17.2)	51.2 (20.3)	<0.0001
Work role (19 items)	60.0 (22.5)	42.5 (19.8)	69.6 (19.8)	61.3 (20.2)	79.3 (13.4)	55.1 (17.6)	<0.0001

Bold is used to highlight the lowest and highest overall competence level between different countries.

1 One-way analysis of variance, SD = standard deviation.Statistically significant differences in overall professional competence between the countries:

2 Finland vs Germany <0.0001, Lithuania <0.0001, Spain 0.0013 and Iceland 0.015.

3 Germany vs Spain <0.0001 and Lithuania 0.0002.

4 Iceland vs Lithuania <0.0001.

5 Lithuania vs Spain <0.0001.

Among the individual items, NMs rated the item ‘Utilising information technology in work’ in the competence category ‘Work role’ as highest in all countries (mean 74.9) as well as in Iceland (82.2), Spain (72.1) and Finland (70.9), indicating good or very good competence. The item ‘Able to recognise situations posing a threat to life early’ in competence category ‘Managing situations’ was rated highest in Germany (81.5), but eighth highest in all countries (64.2). The items ‘Co-ordinating patient education’ (47.1), ‘Updating written guidelines for care’ (48.1) and ‘Evaluating patient education outcomes with family’ (48.5) were rated the lowest, but at a quite good level of competence. Five of the ten lowest scores were in the competence category ‘Therapeutic interventions’ (Table 3).

**Table 3.** Top 10 and bottom 10 items rated by nurse managers (*n* = 425).

*10 highest scoring items* (Meretoja et al., 2004a)	Competence category	All Mean (SD)	Finland Mean (SD)	Germany Mean (SD)	Iceland Mean (SD)	Lithuania Mean (SD)	Spain Mean (SD)
Utilising information technology in work	Work role	74.92 *(19.92)*	70.90 *(20.25)*		82.21 *(16.49)*		72.13 *(19.78)*
Support student nurses in attaining goals	Teaching and coaching	68.13 *(20.78)*					
Taking active steps to maintain and improve professional skills	Teaching and coaching	66.67 *(21.58)*					
Decision-making guided by ethical values	Helping role	66.52 *(21.72)*					
Acting appropriately in life-threatening situations	Managing situations	66.48 *(24.37)*					
Keeping nursing care equipment in good condition	Managing situations	65.42 *(25.63)*					
Committed to the organisation’s care philosophy	Ensuring quality	65.31 *(23.90)*					
Able to recognise situations posing a threat to life early	Managing situations	64.27 *(26.24)*		81.51 *(20.32)*			
Able to recognise colleagues’ need for support and help	Work role	64.21 *(24.19)*					
Making decisions concerning patient care taking the particular situation into account	Therapeutic interventions	64.07 *(23.45)*					
10 lowest scoring items							
Coordinating patient education	Teaching and coaching	47.16 *(25.27)*				62.17 *(24.53)*	
Updating written guidelines for care	Therapeutic interventions	48.13 *(30.37)*					
Evaluating patient education outcomes with family	Teaching and coaching	48.50 *(24.58)*					
Providing consultation for the care team	Therapeutic interventions	48.63 *(30.10)*	29.85 (*23.39)*				
Utilising research findings in nursing interventions	Therapeutic interventions	49.41 *(27.46)*		*42.29 (29.14)*			
Contributing to further development of multidisciplinary clinical paths	Therapeutic interventions	49.71 *(28.38)*					
Utilising nursing research findings in relationship with patients	Helping role	50.27 *(25.07)*					
Making proposals concerning further development and research	Therapeutic interventions	50.50 *(27.14)*					
Developing orientation programmes for new nurses in the unit/ward	Teaching and coaching	50.75 *(26.93)*					
Arranging debriefing sessions for the care team when needed	Managing situations	51.26 *(32.77)*				84.84 (*14.54)*	
Lowest scoring items in a single country							
Mentoring novices and advanced beginners	Work role	53.98 *(32.17)*			21.73 *(22.54)*		
Providing expertise for the care team	Work role	52.48 *(31.48)*					38.68 *(25.14*)

SD: standard deviation.

### Background factors for NMs related to the assessment of NGN’s competence

In an univariate analysis, the country (*p* < 0.0001) was associated with NMs’ competence assessment. Based on multivariate analysis, several background factors of NMs related to competence assessment. NMs gave lower competence assessments if their undergraduate education was a university degree (bachelor; *p* = 0.006), their work title was nurse unit manager (*p* < 0.0001), and NGNs sought to work in manager’s unit very often (*p* = 0.039). As far as NMs’ satisfaction with their current job is concerned, the results are mixed. However, the majority of respondents who were satisfied with their job rated the competence of the NGNs as good. The longer the participants’ work experience as a NM, the higher they rated the competence of the NGNs (*p* = 0.026; Table 4.)

**Table 4.** Associations of background factors with nurse managers’ (*n* = 425) assessments to the overall competence score.

Background factors	Overall score, model based mean estimate/Slope (B)*	95% CI	Univariate *p*-value	Multivariable *p*-value
Individual background factors				
Age	−0.07*	−0.28 to 0.13	0.48	
Gender			0.11	
Female	58.8	56.7 to 61.0		
Male	54.1	48.6 to 59.6		
Undergraduate nursing education			**0.004**	**0.006**
University degree (bachelor)	56.2	53.5 to 58.9		
College/Diploma	58.5	55.4 to 61.6		
Other	69.3	62.2 to 76.4		
Postgraduate degree			0.13	
Doctoral degree	57.7	47.0 to 68.4		
Master’s degree	57.2	54.0 to 60.4		
Bachelor’s degree	62.1	58.4 to 65.9		
No postgraduate degree	56.4	52.8–60.0		
Work experience as a manager	0.34*	0.12 to 0.56	**0.002**	**0.026**
Work experience on the current unit	0.26*	0.06 to 0.46	**0.011**	
Organisational background factors				
Current work title			**<0.0001**	**<0.0001**
Director of Nursing	62.0	52.2 to 71.8		
Nurse Unit Manager	48.6	44.5 to 52.8		
Assistant Unit Manager	60.8	58.0 to 63.0		
Other	60.5	55.1 to 66.5		
Staff report	−0.00*	−0.02 to 0.01	0.64	
NGNs seeking to work in unit			**0.012**	**0.039**
Very seldom	62.2	56.1 to 68.3		
Seldom	61.9	57.6 to 66.2		
Often	57.3	54.6 to 60.0		
Very often	51.9	46.9 to 56.9		
Satisfaction with. . .				
The current orientation programme in unit			**0.015**	
Fully disagree	47.9	35.4 to 60.5		
Almost disagree	51.7	46.0 to 57.5		
Almost agree	58.1	55.5 to 60.8		
Fully agree	47.9	57.8–65.2		
The current job			**0.009**	**0.019**
Fully disagree	75.7	52.9 to 98.5		
Almost disagree	47.5	40.3 to 54.7		
Almost agree	58.4	55.6 to 61.2		
Fully agree	59.9	56.8 to 62.9		
The quality of care in unit			0.51	
Fully disagree	48.8	32.5 to 65.0		
Almost disagree	53.7	43.8 to 63.7		
Almost agree	58.3	55.7–60.8		
Fully agree	59.1	55.7 to 62.5		
The nursing profession			0.27	
Fully disagree	55.1	35.3 to 75.0		
Almost disagree	59.1	51.3 to 66.9		
Almost agree	56.2	53.4 to 59.1		
Fully agree	60.3	57.3 to 63.2		

* These explanatory variables (age, work experience) were included as continuous variables in the model, and therefore the result is expressed as slope.

## Discussion

This multinational European study aims to assess NGNs’ professional competence in the transition phase as perceived by NMs and to identify possible background factors related to their assessments. In the transition phase, the link to nursing education is apparent, and it is also the starting point for NGNs career development. The viewpoint of NMs on NGNs’ professional competence is seldom studied. The goal of this study was to produce knowledge for the cooperation of working life and nursing education to promote the transition and continuous professional competence development of the NGNs.

In this study, NMs assessed the overall professional competence of NGNs with NCS (Meretoja et al., 2004a) as ‘good’, which is in line with previous studies (Numminen et al., 2014; Flinkman et al., 2017). It should be noted, however, that while NMs rated the competence level of NGNs as ‘good’, the competence rating was at the lowest level of the ‘good’ ratings. In Finland, competence was assessed even lower, ‘quite good’, but in Lithuania it was assessed higher, ‘very good’. There might be several reasons for this. One could be differences between the NGNs’ actual achieved competence level and the expected competence level by NMs. NGNs’ legal and professional responsibilities are different from those of nursing students, which may affect the NMs’ competence assessment. Indeed, NMs have found that NGNs face challenges in building relationships with patients while performing nursing tasks (Södersved Källestedt et al., 2020). These challenges may be due to the different situations that NGNs face after graduation, resulting from increased responsibilities and workload, such as the number of patients and more complex care situations that need to be handled independently (Charette et al., 2019a; Södersved Källestedt et al., 2020). Therefore, it is important that students have opportunities to participate in a variety of challenging patient care situations and emergency care already during their nursing education (Kaihlanen et al., 2019; Hyun et al. 2020).

The professional competence of NGNs in all seven competence categories was also on average assessed as good, indicating higher competence level compared to the results of Numminen et al. (2014). However, it is notable that the study of Numminen et al. (2014) included only Finnish participants, and the results for Finland in this study are thus consistent with it. In this study, competence in using information technology at work was rated as good or very good in all countries, which is in line with the results of Numminen et al. (2014). This result is encouraging, as technology is having an increasing global impact on health services in many ways (Bhatia, 2021). The result might be explained by the fact that NGNs are representing younger generations, who are more comfortable using technology (Culp-Roche et al., 2020).

One of the lowest scores was given to the competence category ‘Teaching-coaching’ and a single item on the coordinating patient education. Another study has also identified needs for developing the competence of novice nurses in coaching, supervision and mentoring (Numminen et al., 2014). In nursing education and healthcare, supporting nurses’ skills in patient education is crucial as it correlates with improvements in nursing care quality (Gröndahl et al., 2019) and promotes patient empowerment, which increases patients’ involvement and engagement in their own care (Hickmann et al., 2022).

This study found statistically significant differences in the assessments between countries. Some of the differences may be explained by cultural factors, national legislation and health policies, and variations in the healthcare system. Moreover, the variation in nursing education (Antao et al., 2023; Eronen et al., 2023) may explain the differences despite efforts to harmonise nursing education at the European level (EU, 2005, 2013), and the fact that in all five countries participating in this study, education is delivered in line with the Bologna Process and EU directives (EHEA, 2024; EU, 2005, 2013). This means that nurses may not be able to perform the same tasks (van Kraaij et al., 2023). It is therefore important, in dialogue with employers in the healthcare sector, to continue to develop joint planning, implementation and evaluation of the theoretical and clinical nursing education, as equal competence of nurses will contribute to the quality and safety of patient care and enable the free movement of nurses in Europe.

The variation in nurses’ role, tasks and degree of autonomy in healthcare systems (Gobbi, 2014) might explain the country differences in this study. Organisational factors are reported to influence NGNs’ competence development, such as orientation, stability, workload and the scientific culture of the unit (Charette et al., 2019b) and might increase the demand for NGNs’ level of competence in healthcare systems. For example, in Finland, the number of nurses is one of the highest in Europe (OECD, 2023), and tasks have been transferred from physicians to nurses, including expanded job descriptions and more autonomous roles for nurses. Thus, in some cases, nurses are performing tasks that nurses in other countries do not (Ensio et al., 2019), and NMs may expect NGNs to master these skills as soon as they graduate. As the scientific knowledge base and technical expertise in the nursing profession in Europe has grown, it challenges the minimum standards of education in Europe (Gobbi, 2014). The changing and increasing demands of healthcare mean that nursing education, continuing education and the nursing profession must constantly evolve (van Kraaij et al., 2023).

This study shows that while NMs assessed the competence of the NGNs as good, there is room for improvement. On the other hand, it is unrealistic to expect basic nursing education to produce specific competence that is applicable to all healthcare settings (Numminen et al., 2014). Although nurses themselves have a professional responsibility to maintain their competence after graduation (Bindon, 2017), NMs have a responsibility to ensure that NGNs meet the workplace requirements (Brown and Crookes, 2016; Julnes et al., 2022). NMs also have an important role to play in ensuring the continued competence and career development of NGNs (Gobbi, 2014), as the transition from nursing education to working life is a significant and challenging phase (Duchscher, 2008; Masso et al., 2022). Supporting and retaining NGNs in the workforce is crucial as there is a global shortage of nurses (World Health Organization, 2021; Willman et al., 2022; Brown et al., 2024), and as representatives of employers, NMs should ensure the availability of a competent workforce in the global competition. A recent review (Brown et al., 2024) has identified that for the newcomers, the engagement with the profession and their intention to leave or stay in the profession is related to their ongoing professional development.

There is little research on NMs’ background factors related to the assessment of NGNs competence. In this study, the individual and organisational background of the NMs was related to the assessment. NMs gave lower competence assessments if their undergraduate education was a university degree (bachelor). It is possible that NMs emphasise different areas of competence, such as technical skills, in relation to their own level of education (Chase, 2010), or NMs’ assessments may be based on the expected level of competence in a particular clinical setting (Numminen et al., 2014). Professional competence develops gradually (Wu et al., 2015; Gregg, 2020), so it is important that NMs understand and strengthen the unique abilities of each employee.

This study found that the longer the participants’ work experience as a NM, the higher they rated the competence of the NGNs, which contradicts the study by Numminen et al. (2014). It may be that experienced NMs have a more comprehensive understanding of the NGNs’ competence based on their own experience of performance appraisal and transition programme than novice NMs, as NMs’ own competence develops primarily through experience (Warhaswsky and Cramer, 2019).

The nurse unit managers assessed NGNs’ level of competence lowest in this study. However, the data do not provide an explanation for this. One of the important roles of NMs is to facilitate the professional development of nursing staff, but they face challenges in ensuring sufficient competence and lack the authority to prioritise the professional development of nursing staff (Filkins, 2003; Julnes et al., 2022). Healthcare organisations can support NMs in their role, for example by providing guidelines and tools for nurses’ competence development and assessment (Julnes et al., 2022). For example, regular use of the NCS would appear to be an appropriate tool for monitoring and assessing nurses’ competence and could also be used to identify educational needs and plan further education for nurses (Lejonqvist and Kajander-Unkuri, 2022). Moreover, it is important for healthcare organisations to consider factors that also contribute to NMs’ own job satisfaction and retention (Cox, 2019), as this study indicates that NMs’ satisfaction with their current job was related to their competency assessment. However, the relation was ambiguous and more research is needed.

Research on a comprehensive assessment of the professional competence of NGNs from a managerial perspective is lacking (Kukkonen et al., 2020). This multi-country European study partly fills this gap. Studying professional competence allows a homogenous assessment of how well NGNs’ educational preparation corresponds with nursing practice, as there appear to be different perceptions about NGNs’ level of competence between different stakeholders (Numminen et al., 2014; Hyun et al., 2020). In the future, it is important to ensure that managers at different levels have a common basis and criteria for assessing nurses’ competence, as this study found that the background factors of NMs were related to their assessment.

## Limitations

This study has limitations. Firstly, there are problems in comparing countries, but the common EU rules and labour turnover and shortage made it important to assess the professional competence of NGNs at European level. The curricula in nursing education are relatively homogeneous through EU directives (EU, 2005, 2013), even if there are differences. In addition, the types of nursing education in the participating countries vary, as nurses are trained at university, university of applied sciences or college level. It is important to continue to study different countries in order to find areas for improvement of NGS’ professional competence that should be unified in Europe.

Secondly, despite a large sample of five European countries, with a response rate of almost 70%, the number of participants was heterogeneous and uneven across countries. The background of the NMs varied, and there is inconsistency in the terminology of NMs titles in the countries. However, all respondents worked in management and leadership positions. Comparative research on NMs is scarce internationally and should be increased.

Thirdly, the data were collected with the NCS which is a validated instrument to measure the generic competence of nurses and also developed in relation to managerial perspective (Flinkman et al., 2017). The different jobs and roles of nurses and NMs as well as cultural differences between countries can contribute to NMs having different perceptions of the professional competence of NGNs. Consequently, only tentative conclusions and generalisations of the findings with caution can be made.

## Conclusions

NMs assessed the competence level of NGNs as good to meet the demands of their work in the transition phase, although there are areas for improvement. There are also clear differences between countries, although in all five countries covered by this study, education is provided in line with the Bologna Process and EU directives. It is therefore important to further develop, in cooperation with healthcare employers and educational institutions, the joint planning, implementation and evaluation of theoretical and clinical training for nurses to ensure the quality and safety of patient care and the availability of competent nursing workforce in Europe. Moreover, it is important to continue to study different countries in order to find areas for improvement of NGNs professional competence. The individual and organisational background factors of NMs were related to their assessment. Thus, research is also needed on the relationship between NMs’ background factors and competence assessment. The results can be used for examining the relationship between registered practice and nursing education to promote transition and continuous professional development of NGNs in Europe.

**Key points for policy, practice and/or research**

-  This paper reports multinational assessments of NMs on the professional competence of NGNs, a topic rarely studied.
-  The examining the relationship between registered practice and nursing education to promote transition and ongoing competence and career development of NGNs in Europe, which could facilitate availability of the nursing workforce in the global competition.
-  It is important to further develop, in cooperation with healthcare employers and educational institutions, the joint planning, implementation and evaluation of theoretical and clinical training for nurses to ensure the quality and safety of patient care across Europe.
-  The NCS can be used as tool for monitoring and assessing NGNs’ competence and can also be used to identify and plan further education for nurses.
-  The professional competence of NGNs and related factors from the perspective of NMs need to be further investigated multinationally, using different methodologies, longitudinal study designs and larger samples.
